# Tumor Characterization in Breast Cancer Identifies Immune-Relevant Gene Signatures Associated With Prognosis

**DOI:** 10.3389/fgene.2019.01119

**Published:** 2019-11-12

**Authors:** Jie Li, Cun Liu, Yi Chen, Chundi Gao, Miyuan Wang, Xiaoran Ma, Wenfeng Zhang, Jing Zhuang, Yan Yao, Changgang Sun

**Affiliations:** ^1^College of First Clinical Medicine, Shandong University of Traditional Chinese Medicine, Jinan, China; ^2^Department of Oncology-Pathology, Karolinska Institutet, Stockholm, Sweden; ^3^College of Management, Beijing University of Chinese Medicine, Beijing, China; ^4^Clinical Medical Colleges, Weifang Medical University, Weifang, China; ^5^Department of Oncology, Weifang Traditional Chinese Medicine Hospital, Weifang, Shandong, China; ^6^Innovative Institute of Chinese Medicine and Pharmacy, Shandong University of Traditional Chinese Medicine, Jinan, Shandong, China

**Keywords:** breast cancer, immune-related genes, prognosis, predictive biomarker, characterization

## Abstract

There has been increasing attention on immune-oncology for its impressive clinical benefits in many different malignancies. However, due to molecular and genetic heterogeneity of tumors, the activities of traditional clinical and pathological criteria are far from satisfactory. Immune-based strategies have re-ignited hopes for the treatment and prevention of breast cancer. Prognostic or predictive biomarkers, associated with tumor immune microenvironment, may have great prospects in guiding patient management, identifying new immune-related molecular markers, establishing personalized risk assessment of breast cancer. Therefore, in this study, weighted gene co-expression network analysis (WGCNA), single-sample gene set enrichment analysis (ssGSEA), multivariate COX analysis, least absolute shrinkage, and selection operator (LASSO), and support vector machine-recursive feature elimination (SVM-RFE) algorithm, along with a series of analyses were performed, and four immune-related genes (*APOD*, *CXCL14*, *IL33*, and *LIFR*) were identified as biomarkers correlated with breast cancer prognosis. The findings may provide different insights into prognostic monitoring of immune-related targets for breast cancer or can be served as reference for the further research and validation of biomarkers.

## Introduction

Recent analyses of the single-cell genome and transcriptome of breast cancer (BC) have provided insights into the prognosis between tumors and immune cells (Wagner et al., 2019). Large-scale atlas deepened our understanding of the recognition of tumors and their immune environments in breast cancer ecosystems. The immune system has been shown to be a determining factor in the development and progression of cancer. In fact, many reports have confirmed that immune cells and immune-related genes are attractive targets for regulating cancer progression (Elinav et al., 2013; Topalian et al., 2016; Nagarsheth et al., 2017). Notably, innate and adaptive immune cell infiltration are associated with responses to treatments and clinical outcomes, breast cancers with high immune infiltration to have better prognosis (Atreya and Neurath 2008; Fridman et al., 2012; Fridman et al., 2017; Karn et al., 2017). This suggests that the application of immune-based prognostic features in breast cancer is a potential. Based on this, furthermore, it is also of high priority to consider the collaborative efforts to develop immunobiology research and search for prognostic indicators, so as to bring about the best prognostic evaluation (Nik-Zainal and Morganella 2017; Yates and Desmedt 2017).

Computational techniques applied to a large number of tumor gene expression profiles, based on immunological features, can rapidly provide a broader scale of intratumoral immune landscape (Bindea et al., 2013). Previously, tumor immune infiltration was primarily characterized by tissue-based methods such as flow cytometry and immunohistochemistry (IHC), both of which are limited by the amount of tissue required and the number of cell types assayed simultaneously. Currently, emerging bioinformatics resources are assisting these types of analyses. Large-scale public data with gene expression and clinical information, complete biological databases, and sophisticated high-throughput data analysis methods together provide opportunities for identifying broader prognostic features in breast cancer biology. With these approaches, Li et al. (2017) discovered individualized immune prognostic signature in Early-Stage Non-squamous Non–Small Cell Lung Cancer. Shen et al. (2019) have developed a generalized, individualized immune prognostic signature that can stratify and predict ovarian cancer survival. Besides, in the research of Bindea et al. (2013), *CXCL13* and *IL21* are likely associated with long-term survival of patients and absence of tumor recurrence in human colorectal cancer (CRC).

**Figure 1:**
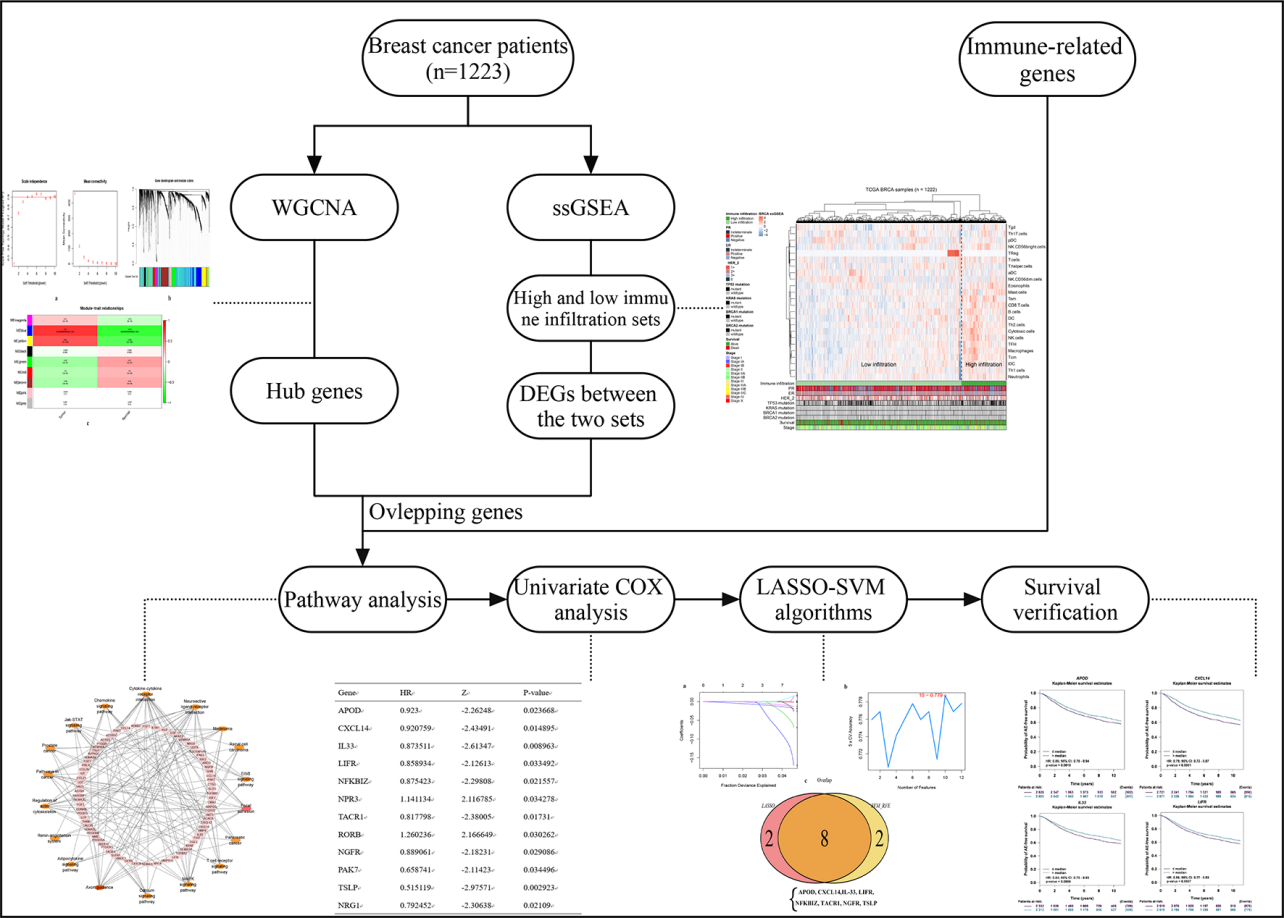
The workflow of the study.

Previous studies have revealed the relationship between immune cell levels and breast cancer prognosis, therefore, on this basis, the current study aimed to assess the association between immune infiltration and the genome in BC to reveal the effect of immune-relevant genes on the prognosis of breast cancer. Specifically, we explored the relationship of immune infiltration (measured by the expression characteristics of immune genes) in molecular level rather than cellular level with patients’ prognosis. In this study, we performed a multi-perspectives, multi-dimensional analysis of a large number of breast cancer samples using massive bioinformatics and machine learning methods, such as weighted gene co-expression network analysis (WGCNA), single-sample gene set enrichment analysis (ssGSEA), differential analysis, univariate COX analysis, least absolute shrinkage and selection operator (LASSO), and support vector machine-recursive feature elimination (SVM-RFE) algorithm, functional analysis, and prognostic verification. Finally, four biomarkers of breast cancer were identified that were thought to be probably important prognostic features in breast cancer. The workflow is showed in **Figure 1**.

## Materials and Methods

### Data Collection and Preprocessing

RNA-sequencing data, updated clinical data, and sample information of breast invasive cancer (BRCA) cohort were downloaded from the TCGA data portal (https://portal.gdc.cancer.gov/); fragments per kilobase million (FPKM) values were transformed into transcripts per kilobase million (TPM) values. Immune-related genes were collected from the ImmPort database (http://www.immport.org) and by tracking Tumor Immunophenotype (http://biocc.hrbmu.edu.cn/TIP/index.jsp); 24 immune cell type-specific gene signatures were adopted from Bindea et al. (2013).

### Definition of BC-Related Genes by Weighted Gene Co-Expression Network Analysis (WGCNA)

First, Cluster 3.0 was used to remove the discrete values of transcripts with less than 50% expression, and differential expressed analysis was performed by R package limma (logFC ≥ 2 or < −0.5 and p-value < 0.05). Pearson’s correlation analysis of the top 4,000 genes, based on standard deviation, was performed to construct a matrix of similarity (Ivliev et al., 2010). According to the power value which main affects the independence and the average degree of connectivity (k) of the co-expression modules, an adjacency matrix (AM) and a Topological overlap matrix (TOM) are obtained. The gradient method was used here and the power values ranged from 1 to 10. When the correlation between k and p (k) reached 0.8, the optimal power value was determined to construct a scale-free topology network. Then TOM that measured the network connectivity of genes was transformed from AM (Botia et al., 2017). Modules were defined as branches of the hierarchical cluster tree generated based on TOM dissimilarity (Langfelder and Horvath 2008). For any given module, since the module Eigengenes (ME) offered the most appropriate interpretation of gene expression profile, we associated the ME with clinical features that refer to tumor or normal status in this study. Modules displaying high correlation (according to Module-trait relationships) were selected as further research objects, and genes of which were named BC-related genes.

### Implementation of Single-Sample Gene Set Enrichment Analysis (ssGSEA)

Related infiltration and activity levels for 24 immune cell types, obtained from published signature gene lists across all tumor and normal samples, were quantified using the ssGSEA in R package GSVA (Hanzelmann et al., 2013). The signatures used in this study comprised of innate immunity, including natural killer (NK) cells, CD56dim NK cells, CD56bright NK cells, dendritic cells (DCs), plasmacytoid dendritic cells (pDC), immature DCs (iDC), activated DCs (aDC), neutrophils, mast cells, eosinophils, and macrophages, and adaptive immunity, including B cells, T cells, T central memory cells (Tcm), T effector memory (Tem), CD8 T cells, cytotoxic cells, T follicular helper (TFH), Th1, Th2, Th17, and Treg cells. The ssGSEA scores for each individual immune cell type were standardized, and the qualitatively different tumor microenvironment cell infiltration patterns were grouped into high-infiltration and low-infiltration ones using hierarchical agglomerative clustering based on Euclidean distance and Ward’s linkage. To identify genes associated with tumor microenvironment cell infiltration patterns, we calculated the differential expressed genes (DEGs) in the two sets using limma package.

### Pathway Enrichment Analysis for the Molecular Function

Overlapping ones among the immune-related genes, BC-related genes and DEGs in high-infiltration versus low-infiltration groups, were subjected to perform enrichment function analysis using R package clusterProfiler to assess the molecular function.

### Feature Selection by Least Absolute Shrinkage and Selection Operator (LASSO) and Support Vector Machine-Recursive Feature Elimination (SVM-RFE) Algorithms

Univariate regression analysis was performed on overlapping genes obtained in *Section Implementation of Single-Sample Gene Set Enrichment Analysis (ssGSEA)* to select the survival-related genes. SVM-RFE and LASSO logistic regression were used to screen the characteristic genes. The LASSO algorithm was applied with the glmnet package (Friedman et al., 2010). Furthermore, SVM-RFE is a machine learning method based on support vector machine, which is used to find the best variables by deleting SVM-generated eigenvectors. SVM module was established to further identify the diagnostic value of these biomarkers in BC by e1071 package (Huang et al., 2014). Ultimately, we combined the genes from either LASSO or SVM-RFE algorithm for further analysis. A two-sided P value < 0.05 was considered to be statistically significant.

### Verification of Survival Prediction

Next, we used external data to validate the survival results in Breast Cancer Gene-Expression Miner v4.2 (Jezequel et al., 2012), a database based on a large number of published studies. Kaplan–Meier survival curves with 2-sided log-rank test were drawn to evaluate OS differences between the two groups divided based on the median quantile expressions of genes.

### GO Analysis of Single Gene

We carried out a holistic GO analysis of the screened biomarkers through the clusterProfiler package. Further, in order to understand the potential molecular function of each biomarker more specifically, guilt by association approach was used in this study, which means that the function of biomarkers could be inferred by performing a functional analysis of the genes that were significantly correlated with biomarkers (Oliver 2000). Pearson’s correlation coefficient threshold was >0.5.

## Results

### Data Downloading and Collection

A total of 1,222 specimens, consisting of 1,109 cancer samples and 113 normal samples, were obtained from TCGA. A total 2,211 immune-related genes were collected from ImmPort database, TTI database, and from the report by Bindea et al. (2013).

### Determination of the Most Relevant Module Genes for BC

**Figure 2:**
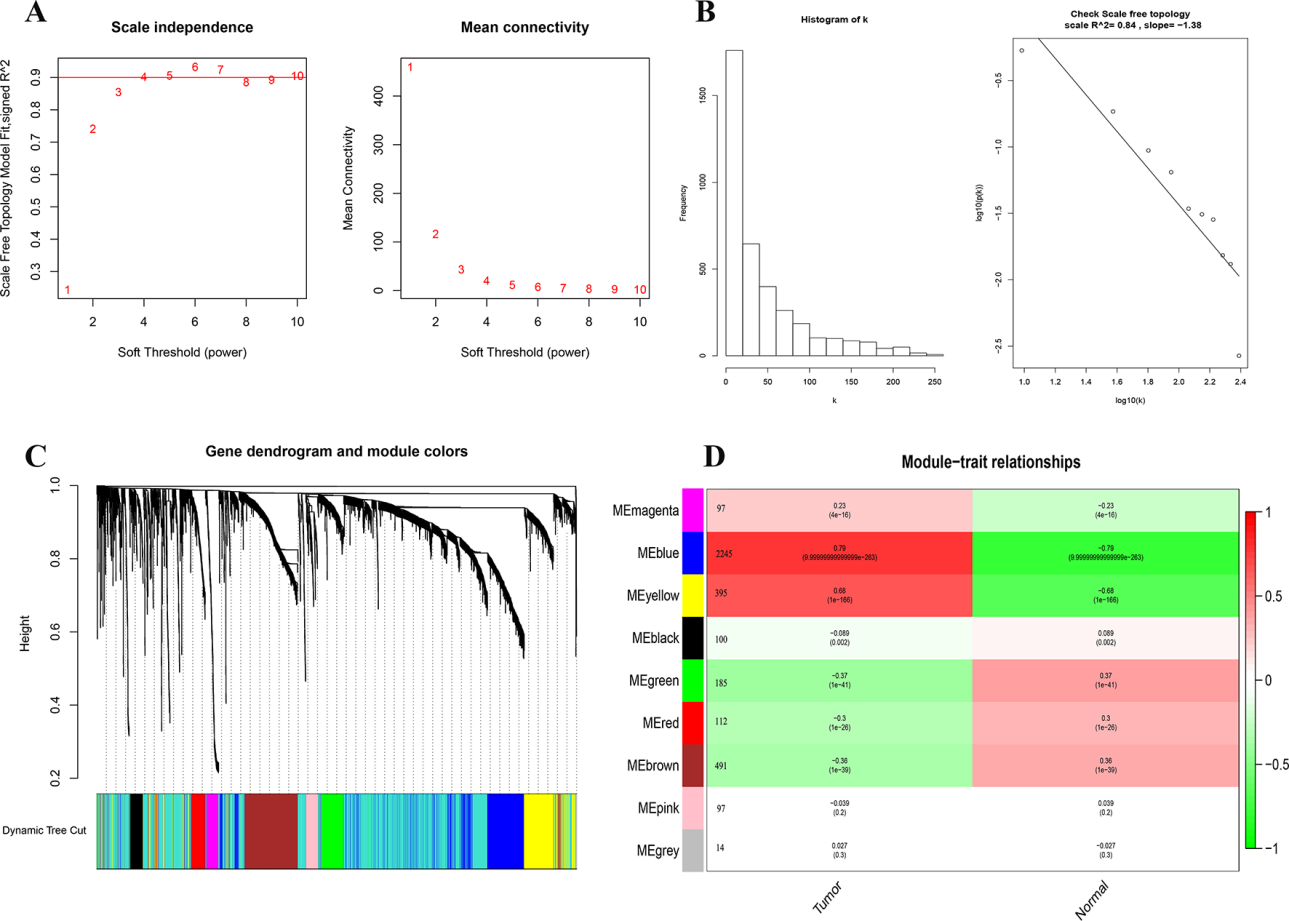
Identification of modules associated with the clinical status of breast cancer in the WGCNA. **(A)** Analysis of the scale-free fit index and the mean connectivity for various soft-thresholding powers. **(B)** Dendrogram of all differentially expressed genes clustered based on a dissimilarity measure (1-TOM). **(C)** Heatmap of the correlation between module Eigengenes and clinical status (normal and tumor), number after the module name is the number of genes in the module. **(D)** Checking the scale free topology when β = 4. K represents the logarithm of whole network connectivity, p(k) represents the logarithm of the corresponding frequency distribution. K is negatively correlated with p(k) (correlation coefficient = 0.84), which represents scale-free topology.

We first identified 5,058 differential expressed genes in 1,222 samples, and selected the top 4,000 (of 4,385) after sorting by standard deviation. Co-expression analysis was performed to construct a co-expression network. A total of 9 modules were identified *via* the average linkage hierarchical clustering. To achieve scale-free co-expression network, power of β = 4 was selected; to merge the highly similar modules, we chose a cut line of < 0.25 and minimum module size of 50, using the dynamic hybrid tree cut method. Blue and yellow modules (Module–trait relationships = 0.79 and 0.68, respectively) were found to have the highest association with tumor status (**Figure 2**), and hence, 2,629 genes in the two modules were considered to be significant module genes for further analysis. Notably, our research differs from other studies about breast cancer using WGCNA method in that our aim is to apply WGCNA to discover hub genes related to immune.

### High-Immune Infiltrated Genes Identified by ssGSEA

**Figure 3:**
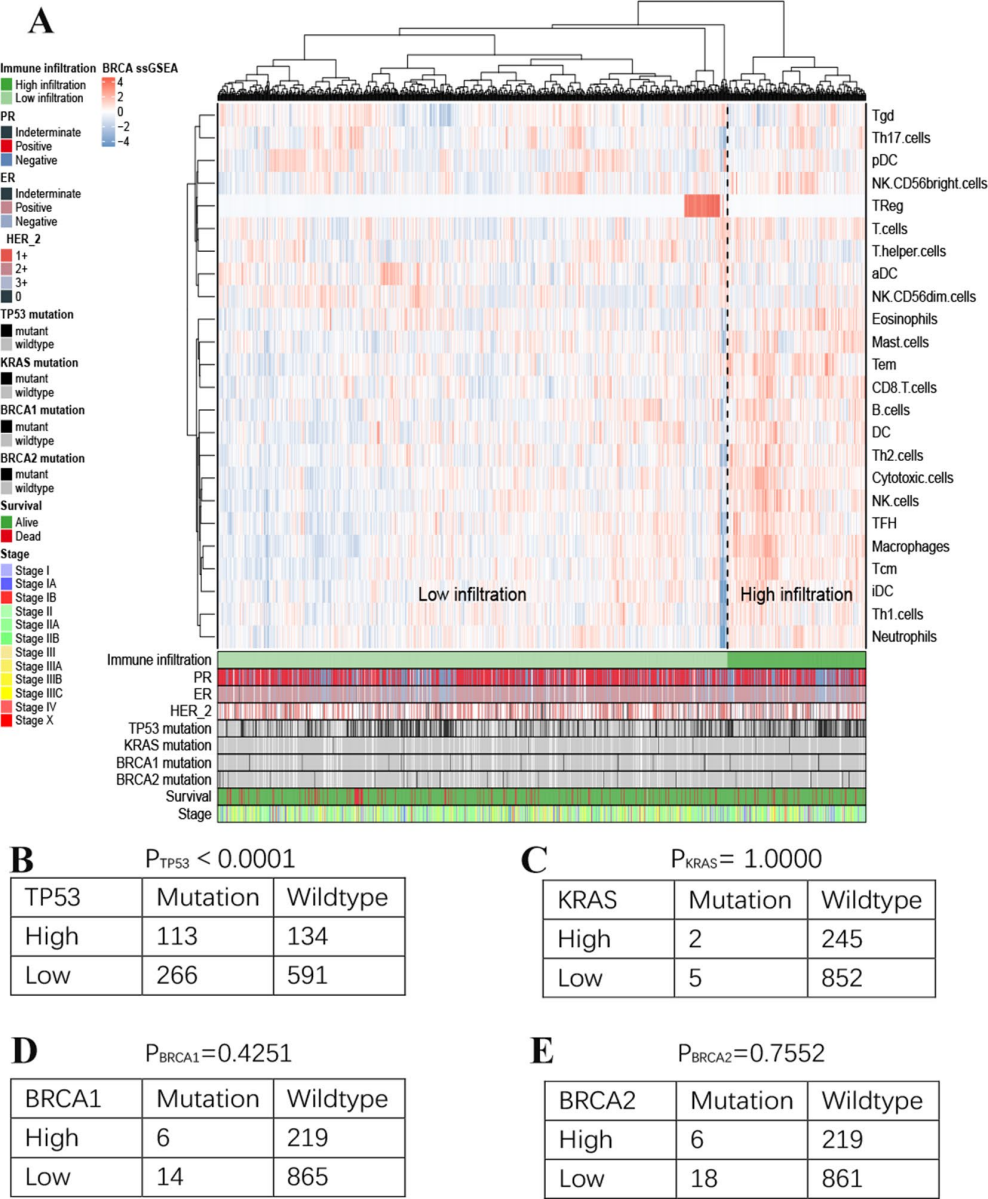
Immune landscape of breast cancer **(A)**. Unsupervised clustering of 1,222 patients from TCGA cohort using single-sample gene set enrichment analysis scores from 24 immune cell types. Two distinct immune infiltration clusters, here termed high infiltration and low infiltration, were defined. The relationship between immune infiltration and *TP53* **(B)** *, KRAS* **(C)** *, BRCA1* **(D)** and *BRCA2* **(E)** mutation status.

ssGSEA analysis was used to assess the immune infiltration level of every gene set, for every sample, by calculating separate enrichment scores for each sample and genome. We used ssGSEA scores from 24 immune-related gene signature extension groups to perform unsupervised clustering on breast cancer samples with transcriptome analysis data and clinical features (**Figure 3A**). This analysis clearly revealed two different clusters referred to herein as the high immune infiltration and low immune infiltration groups. Interestingly, TReg group are significantly different from the other groups. It showd that TReg has less quantified infiltration levels than other immune cells, so the calculated enrichment score in many samples tends to zero. However, focal point of our study is not on immune cells, so we are only focusing on their high and low infiltration groups divided and calculated by these immune feature genes of immune cells here. For further characterization, we performed differential expressed analysis of the genes in high versus low immune infiltration, 2,951 genes were obtained and considered to be potentially associated with tumor immune microenvironment and prognostic effects probably.

In addition, we assessed the association between immune cell infiltration and the mutation status of *TP53, KRAS, BRCA1* and *BRCA2* (**Figures 3B–E**). Significant difference was observed in *TP53* (H = 113; L = 266 vs H = 134; L = 591, *TP53-*mutant vs wildtype; p< 0.001). However, there was no relationship between other mutation status and immune cell infiltration.

### Functional Annotation and Analysis

**Figure 4:**
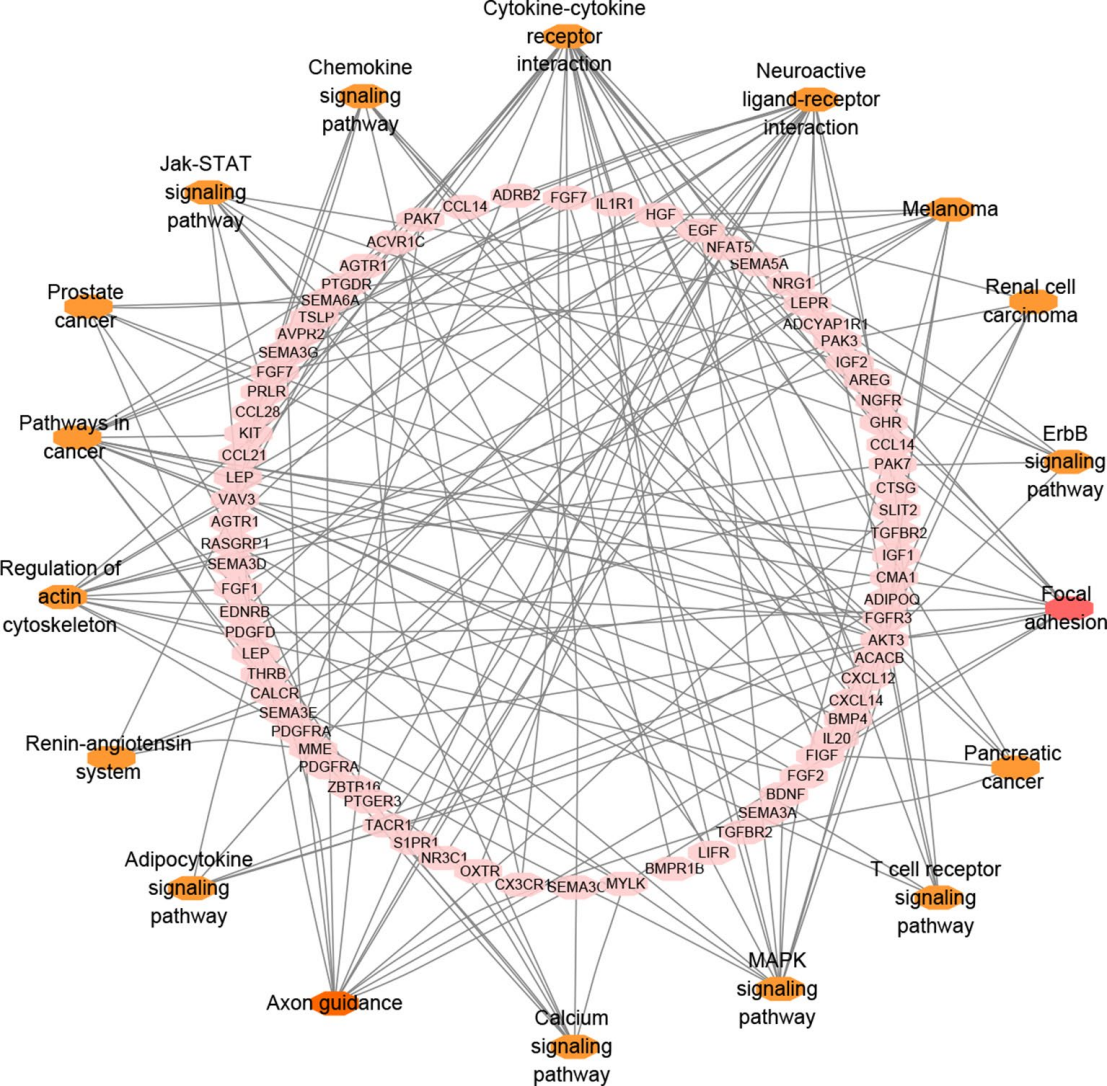
Pathway analysis network of 131 preliminary potential genes.

In total, 131 genes overlapped among BC-related genes, immune-related genes and differential immune infiltrated genes. To improve biological understanding of the genes identified in this study, we conducted enrichment analysis. As shown in **Figure 4**, some pathways like cytokine-cytokine receptor interaction, neuroactive ligand-receptor interaction, and other signaling pathways, were all quite significant in key cascades in the basic biology of cancer, such as initiation, growth, and recurrence of breast cancer (Hanahan and Weinberg 2011; Dees et al., 2012). Interestingly, “MAPK signaling pathway” has been reported to be essential for cancer-immune evasion in human cancer cells (Sumimoto et al., 2006). Moreover, there were many other pathways involved in specific cancers, such as in melanoma, pancreatic cancer, and renal cell carcinoma, many of which have shown optimistic response on immunotherapy.

### Preliminary Identification of Optimal Prognostic Biomarkers

**Figure 5:**
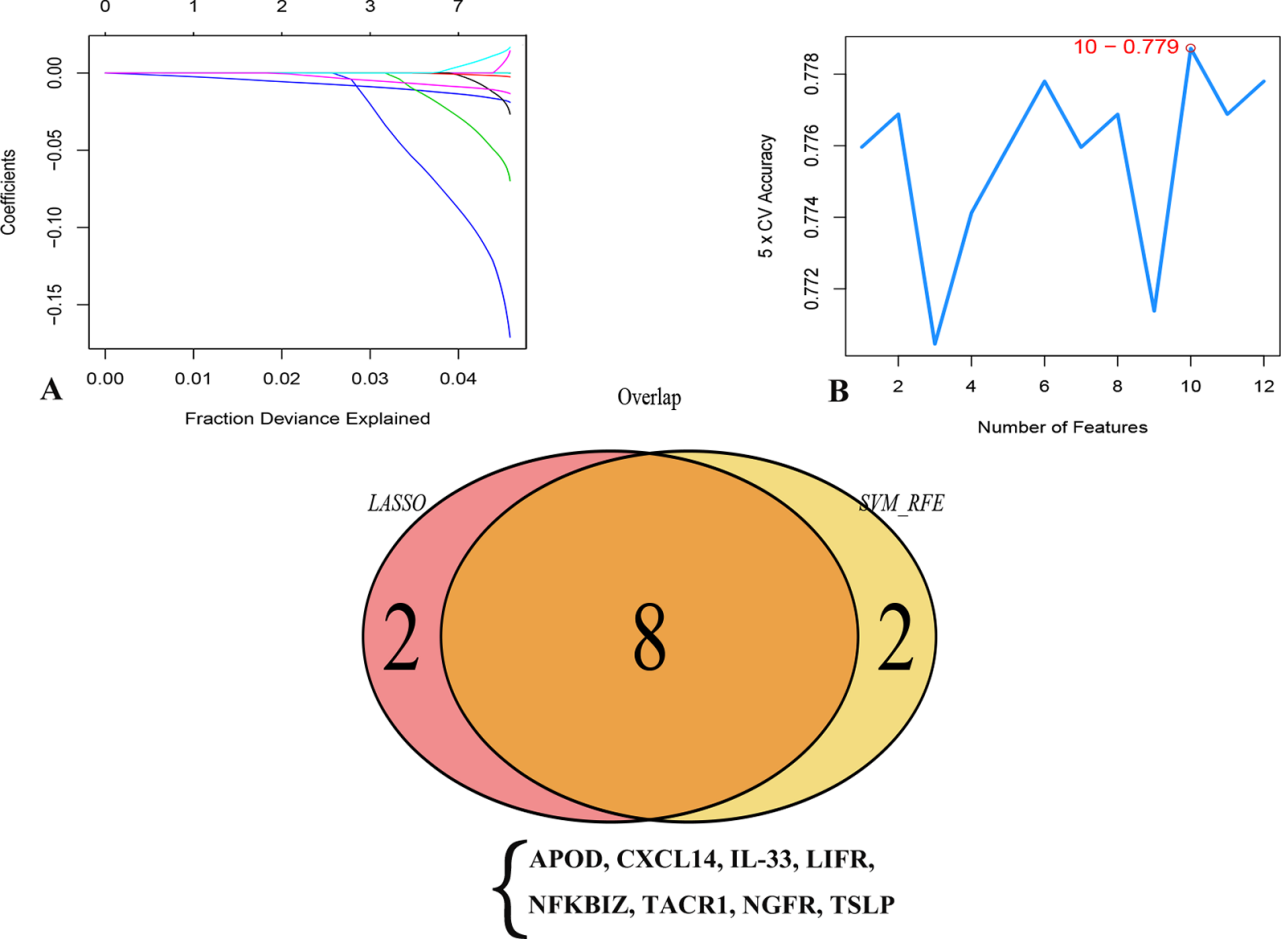
Two algorithms were used for feature selection: LASSO **(A)** and SVM-RFE **(B)** algorithms. **(A)** LASSO coefficient profiles of the 12 genes that met the prognostic criteria initially. **(B)** The point highlighted indicates the lowest error rate, and the corresponding genes at this point are the best signature selected by SVM.

In an attempt to confirm the independent prognostic impact of individual genes, we performed univariate COX regression of the 131 previously screened variables, and obtained 12 genes that met the prognostic criteria (**Table 1**); 10 genes were identified with the LASSO and SVM algorithms, respectively. Eight shared biomarkers for BC were defined by overlapping the biomarkers derived from these two algorithms (**Figure 5**). These 8 genes, associated with the prognosis of BC, are considered to be the best features, and included apolipoprotein D (*APOD*), Chemokine (*C-X-C* motif) ligand 14 (*CXCL14*), Interleukin-33 (*IL33*), leukemia inhibitory factor receptor (*LIFR*), nuclear factor kappa B inhibitor zeta (*NFKBIZ*), tachykinin-1 receptor (*TACR1*), nerve growth factor receptor (*NGFR*), and thymic stromal lymphopoietin genes (*TSLP*).

**Table 1:** The Univariate COX analysis of the signature.

Gene	HR	Z	P-value
*APOD*	0.923	−2.26248	0.023668
*CXCL14*	0.920759	−2.43491	0.014895
*IL33*	0.873511	−2.61347	0.008963
*LIFR*	0.858934	−2.12613	0.033492
*NFKBIZ*	0.875423	−2.29808	0.021557
*NPR3*	1.141134	2.116785	0.034278
*TACR1*	0.817798	−2.38005	0.01731
*RORB*	1.260236	2.166649	0.030262
*NGFR*	0.889061	−2.18231	0.029086
*PAK7*	0.658741	−2.11423	0.034496
*TSLP*	0.515119	−2.97571	0.002923
*NRG1*	0.792452	−2.30638	0.02109

### Survival Verification by External Data

**Figure 6:**
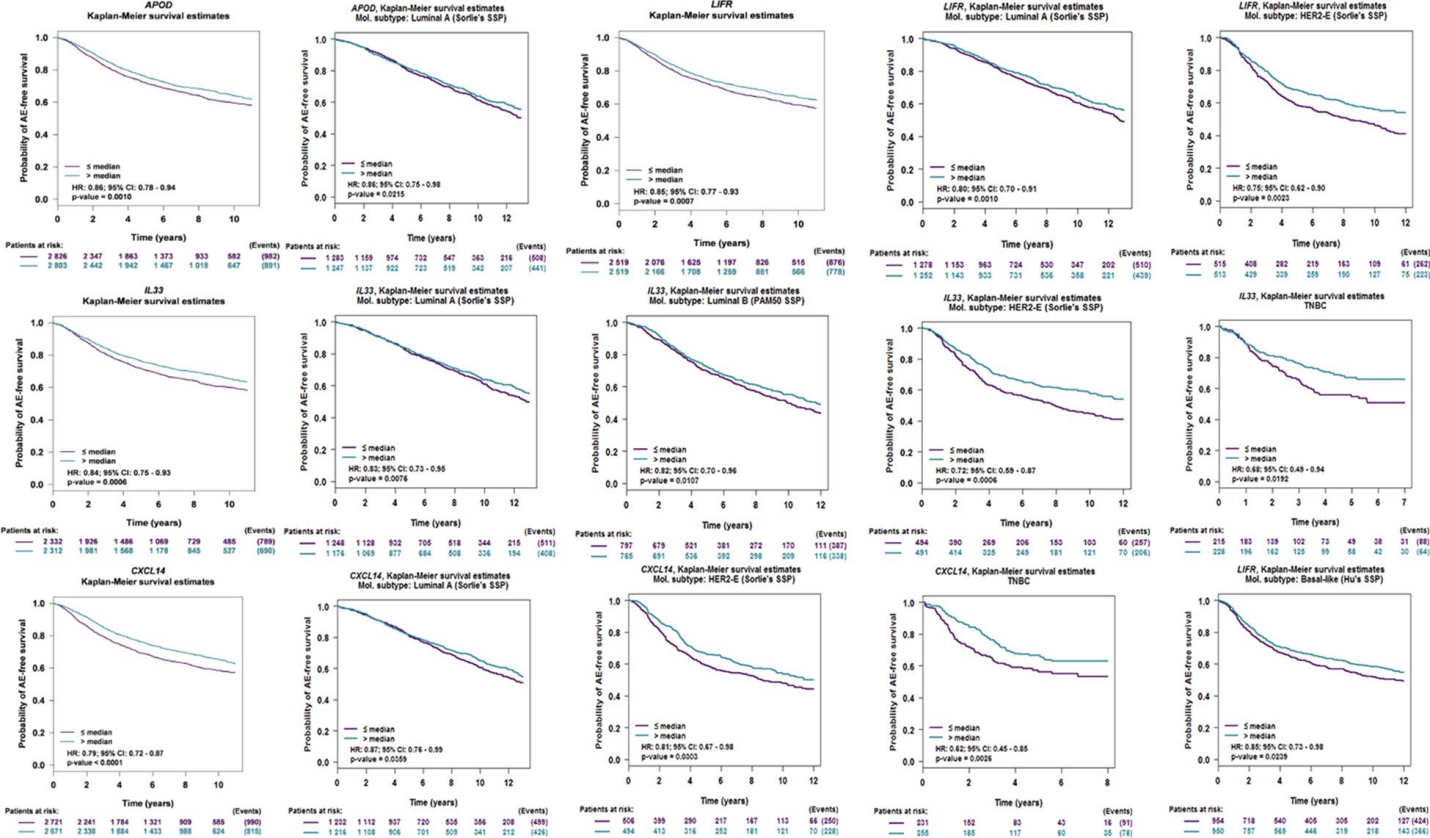
Overall survival of the four potential biomarkers in breast cancer, Luminal A, Luminal B, HER-2 positive breast cancer and TNBC based on Kaplan–Meier-plotter. The patients were stratified into high-level group and low-level group according to median expression.

We put the 8 genes into a web-site (bc-GenExMiner V 4.1) to perform survival analysis. Based on the validation of larger external sample data, our results showed that *APOD*, *CXCL14*, *IL33*, and *LIFR* displayed good prognostic significance (**Figure 6**), Further, we performed prognostic analysis of breast cancer subtypes, and the results indicated that *APOD* shows good prognostic value in luminal A breast cancer, *IL33* is related to prognosis of Luminal A, Luminal B, HER-2 positive breastV cancer and Triple negative breast cancer (TNBC). *CXCL14* and *LIFR* are associated with the prognosis of Luminal A, HER-2 positive breast cancer and TNBC. Therefore, we used these 4 genes as targets for further analyses.

### Potential Significance of the Biomarkers Identified

To ascertain the possible mechanisms of genes affecting BC progression, GO analysis of each biomarker was conducted separately. Molecular functions of the four genes are mainly related to the production, binding, or migration of chemokines, lymphocytes, cytokines, and receptors, which are closely associated not only with the growth process of cancer cells, but also with the immune environment.

## Discussion

Although breast cancer is not considered to be a particularly immunogenic tumor compared to melanoma and renal cell carcinoma, molecular profiling of breast tumors suggests that it does show certain levels of immunoregulatory gene activation (Ascierto et al., 2012). Multiple investigators have reported that immune-related genes can be used as prognostic indicators for breast cancer (Iwamoto et al., 2011; Gingras et al., 2015). The increasing body of evidence demonstrating the importance of biomarkers, especially genes, in determining cancer outcomes provides new opportunities to integrate this information into therapeutic algorithms (Li et al., 2017).

Previous studies about tumors and immune infiltration mostly focused on immune cell types. For example, Li B et al. (2016) infers the abundance of the six immune cell types (B cells, CD4 T cells, CD8 T cells, neutrophil, macrophage, and dendritic cells) using approach of constrained least squares fitting and found many significant associations between immune cell abundance and outcome of 23 cancer types patients. For instance, except for association with prolonged survival of patients, CD8 T cells may also play an important role in preventing tumor recurrence (in melanoma and colorectal cancer and cervical cancer). Notably, the focus of our research is to estimate the degree of 24 immune cells infiltration using ssGSEA, and finally determined the four most promising immune-related genes associated with prognosis.

The original design of this study was to use various bioinformatics tools and databases to identify useful and potential immune-related targets for breast cancer associated with prognostic outcomes. Based on that, in this study, ssGSEA was implemented to calculate the immune cell infiltration levels for each sample in order to determine the two infiltration groups. Since previous studies had shown immune-infiltration to have better prognosis in breast cancer, we analyzed the differential genes in the high and low groups. To identify the genes most relevant to breast cancer, we performed a WGCNA analysis to select the modules with the strongest correlation between the module traits and genes in the modules. At the same time, we collected immune-related genes from various databases and literatures; the overlapping 131 genes of the above three clusters were identified as the immune strongly related genes associated with breast cancer. Functional analysis showed these 131 genes to be closely associated with the development of breast cancer and immune escape, such as *via* cytokine–cytokine receptor interaction and MAPK signaling pathway. Next, unlike previous studies that selected markers only by one algorithm, our current study chose a combination strategy to minimize the possibility of losing important markers through incorporating genes from three distinct algorithms. Based on univariate regression analysis determining the genes associated with prognosis, LASSO and SVM-RFE algorithms were performed to screen eight characteristic variables. After extensive survival validation tests with external data, four of eight genes (*APOD*, *CXCL14*, *IL33*, and *LIFR*) were successfully identified as prognostic markers and the other four were filtered out because they did not show good prognostic differences in validation.

Besides, we tested whether the infiltration of immune cells is associated distinct mutations, and the results indicated that *TP53* showed relatively obvious difference between the mutant and the wild type. These differences may illustrate that *TP53* mutation may affect the immune microenvironment of breast cancer to a certain extent (the specific mechanism is not clear here), this is consistent with the conclusion of a recent study (Liu Z et al., 2019).

Further, functional analysis of the four identified biomarkers showed them to be mainly related to the production, metastasis, or regulation of chemokines, cytokine–cytokine interaction, lymphocytes, and leukocytes, which contribute to the progress of cancer and immunity. Specifically, chemokines and cytokines can lead to recruitment of multiple cell types, including different immune cell subsets, into tumors, thereby involving in angiogenesis, inflammation, tumor growth, invasion, metastasis, and anti-tumor immunity (Motz and Coukos 2011).

*CXCL14* (earlier designated as *BRAK*, *MIP-2γ*, *BMAC*, or *KS1*), was identified in this study as one of the chemokines that had been previously shown to play a key role in breast cancer. For example, in the animal models of Allinen et al. (2004) *CXCL14* was shown to be overexpressed in tumor myoepithelial cells; it binds to receptors located on epithelial cells and increases the proliferation, migration, and invasion of BC. However, in patients, *CXCL14* tends to show a more favorable correlation between protein level and overall BC survival without considering the cell type in charge of *CXCL14* expression, as was consistent with our findings (Gu et al., 2012). In addition, some studies have suggested that it displays chemotactic activity towards various immune cells, including B-cells, NK cells, and monocytes (Shellenberger et al., 2004). This also consolidates and complements our functional analysis results. *IL33* is a member of the IL1 cytokine family, and has been suggested to play a pro-tumoral role in BC. For example, several research found that *IL33* facilitates the expansion of immunosuppressive myeloid, ILC2, and Treg cells, and reduces activated NK cells, thereby enhancing the growth of BC, and development of lung and liver metastases (Xiao et al., 2016; Afferni et al., 2018). Another study pointed that high *IL33* value in 4T1 breast cancer cells reduces tumor growth and metastasis *in vivo* (Jovanovic et al., 2014).

Our findings are particularly notable considering that recent studies have reported *LIFR*, the receptor of leukemia inhibitory factor (LIF), as a prognostic marker of BC for clinical outcomes. This protein is upregulated in normal breast tissues, but its high expression in tumor tissues has inverse association with lymph node metastasis. Notably, down regulation or loss of *LIFR* is related to poor prognosis in most nonmetastatic stage I–III breast cancer (Chen et al., 2012). *APOD* is a member of the lipocalin family of proteins, seen in patients with BC; analysis of the relationship between *APOD* expression and clinical outcome demonstrates significant relevance of the low expression of *APOD* with a poor overall survival (Diez-Itza et al., 1994). In previously two small-scale studies of Soiland H et al., the association between *APOD* and recurrence is only found in specific age groups, especially disease-specific survival in elderly, comorbid patients not receiving chemotherapy (Soiland et al., 2009a; Soiland et al., 2009b). However, in a large study, the results of Soiland H et al. could not be verified. At the same time, Klebaner et al. (2017). believe that the true association between *APOD* expression and recurrence may be ineffective in ER+ breast cancer patients treated with tamoxifen. Our study did not consider the patients’ different treatment, and it showed *APOD* expression has a good prognosis value in Luminal A breast cancer. This also suggests that all the markers including *APOD* may need to be further explored in patients receiving different treatments.

Interestingly, there is no report of *IL33* as a prognostic marker of BC, but consistency of our findings regarding *CXCL14*, *LIFR*, and *APOD* with previous studies indicates our method to be reliable, and thus supports the reliability of our conclusions to a large extent.

At present, there are several treatment methods for targeting tumor-associated immune genes, and have achieved good results in clinical or clinical trial. For example, blocking macrophage chemokines such as CXCL12, and preventing macrophages from entering tumors, or fighting for macrophages to kill tumor cells (Gholamin and Mitra 2017). Although focal point of our research is not on the mechanism of action of immune cells, it adds strong evidence that tumor-associated immune genes may become potential targets for cancer therapy. Previous studies have been based on specific layers of immune cells such as tumor infiltrating lymphocytes or developing prognostic models. Our study focuses on immune-related genes and uses strict standard layer screening to obtain genes which may be potential prognosis targets of BC. But our research also has certain limitations, clinical trials of checkpoint inhibitors have indicated immune infiltration to be critical for tumor regression, and that the quality of immune response is a key factor for successful treatment, so substantial clinical trials still need to be carried out.

In conclusion, using bioinformatics and machine learning methods, we focused on immune-related genes of breast cancer based on large data of real samples, and screened four potential prognostic targets, three of which were confirmed in previous studies. Although the four prognostic markers identified in our current study may still need a lot of clinical trials for validation, they may provide some clues and landscape for the prognosis assessment of breast cancer.

## Data Availability Statement

Publicly available datasets were analyzed in this study. The data can be found in https://portal.gdc.cancer.gov/, http://www.immport.org, http://biocc.hrbmu.edu.cn/TIP/index.jsp.

## Author Contributions

JL and CS conceived of the presented idea. CL, XM, and WZ contributed to data collection in consultation with JL. CG, JZ, and YY carried out the analysis. JL and CS wrote the manuscript. YC and MW contributed to the manuscript revision. All authors discussed the results and contributed to the final manuscript.

## Funding

This work is supported by the grants from National Natural Science Foundation of China (81673799) and National Natural Science Foundation of China Youth Fund (81703915).

## Conflict of Interest

The authors declare that the research was conducted in the absence of any commercial or financial relationships that could be construed as a potential conflict of interest.
